# Supplementary material for: Pharmacological EZH2 inhibition combined with retinoic acid treatment promotes differentiation and apoptosis in rhabdomyosarcoma cells
Source: Clin Epigenetics. 2023 Oct 19;15:167. doi: 10.1186/s13148-023-01583-w (PMC10588044; doi:10.1186/s13148-023-01583-w)
Supplement: Supplementary file 2 — Additional file 2. Supplementary Figures. [file 13148_2023_1583_MOESM2_ESM.ppt]

## Slide 1
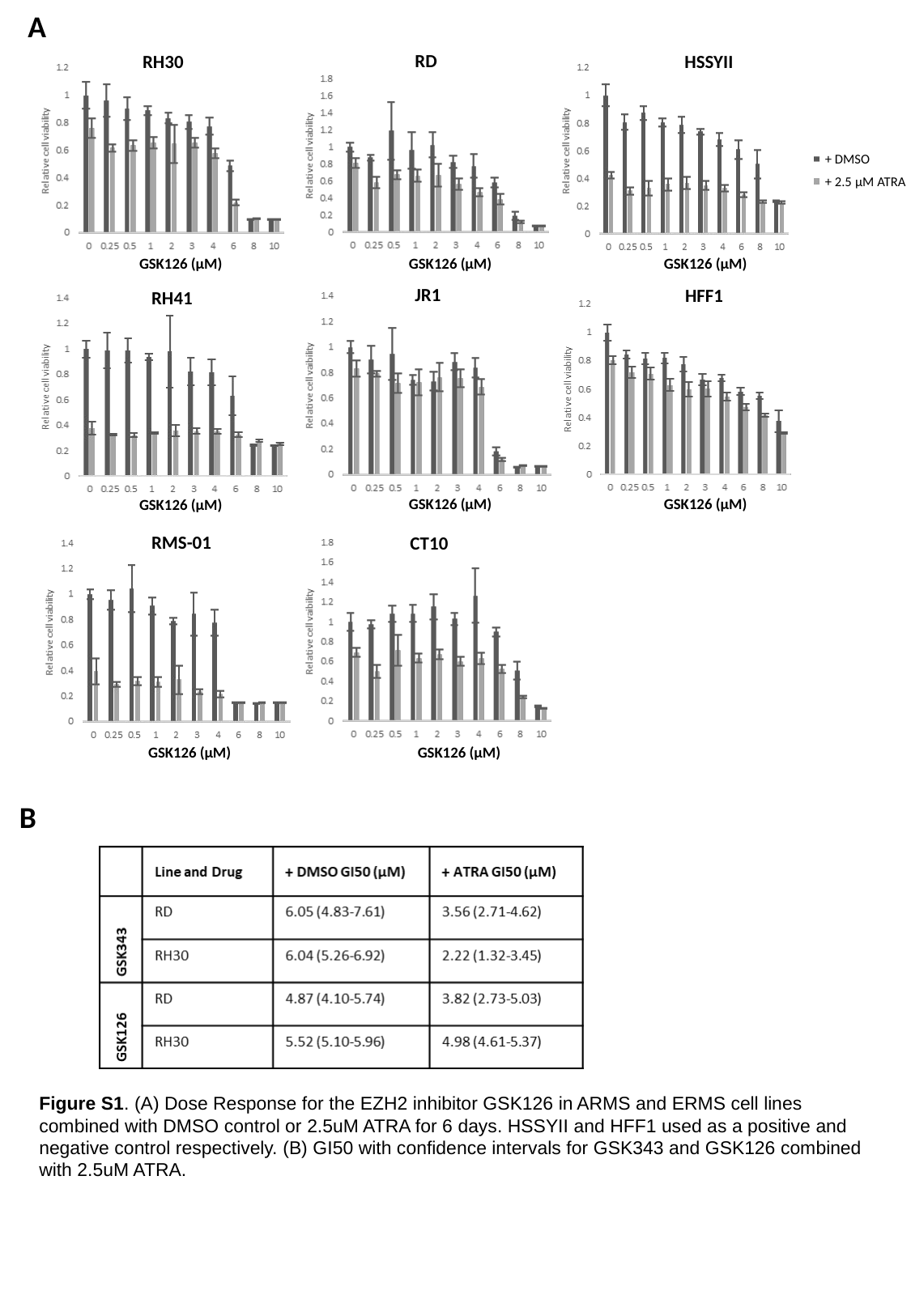

A
RD
RH30
HSSYII
+ DMSO
+ 2.5 µM ATRA
GSK126 (µM)
GSK126 (µM)
GSK126 (µM)
JR1
HFF1
RH41
GSK126 (µM)
GSK126 (µM)
GSK126 (µM)
RMS-01
CT10
GSK126 (µM)
GSK126 (µM)
B
Figure S1. (A) Dose Response for the EZH2 inhibitor GSK126 in ARMS and ERMS cell lines combined with DMSO control or 2.5uM ATRA for 6 days. HSSYII and HFF1 used as a positive and negative control respectively. (B) GI50 with confidence intervals for GSK343 and GSK126 combined with 2.5uM ATRA.

## Slide 2
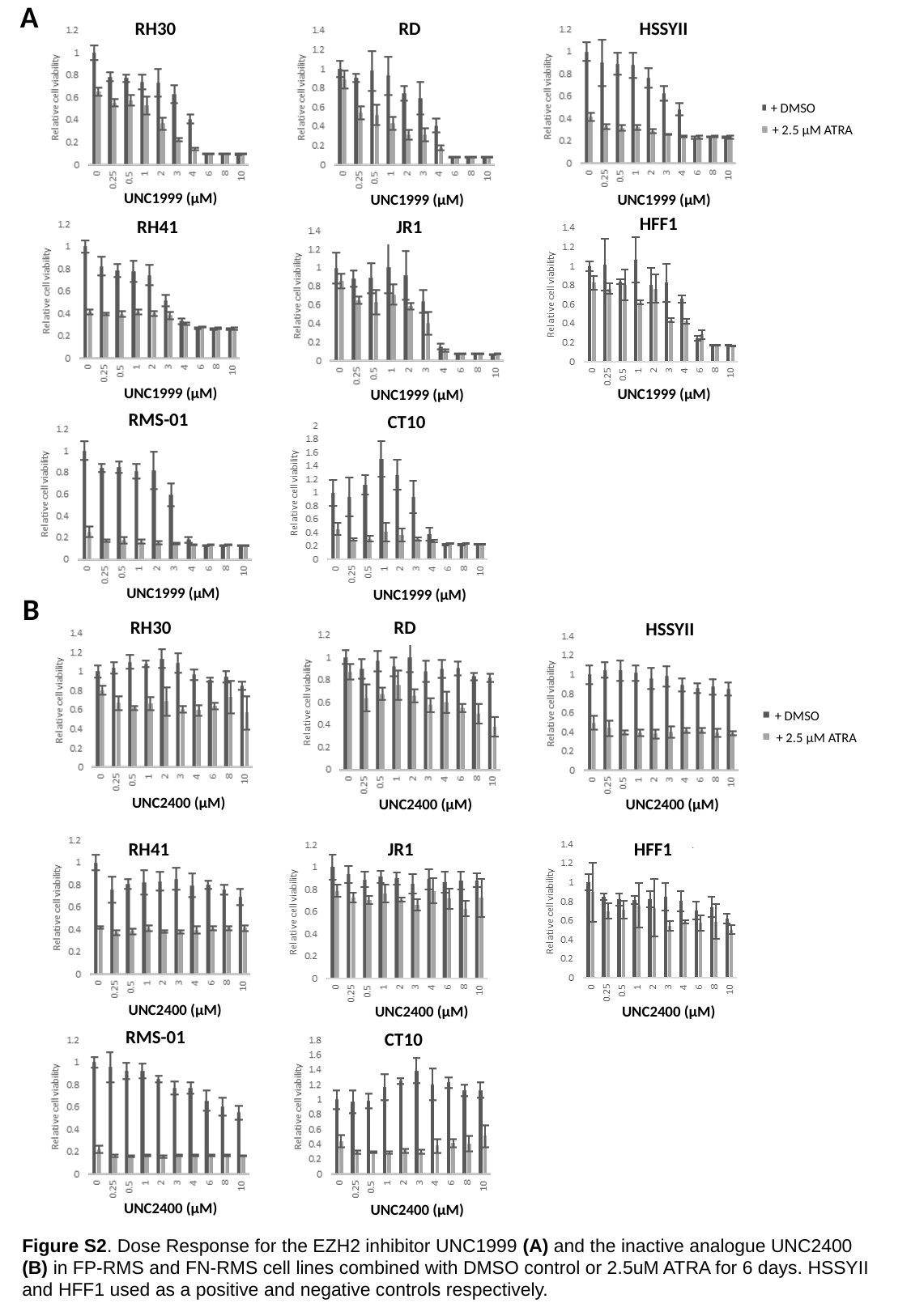

A
RH30
RD
HSSYII
+ DMSO
+ 2.5 µM ATRA
UNC1999 (µM)
UNC1999 (µM)
UNC1999 (µM)
RH41
JR1
HFF1
UNC1999 (µM)
UNC1999 (µM)
UNC1999 (µM)
RMS-01
CT10
UNC1999 (µM)
B
UNC1999 (µM)
RH30
RD
HSSYII
+ DMSO
+ 2.5 µM ATRA
UNC2400 (µM)
UNC2400 (µM)
UNC2400 (µM)
RH41
JR1
HFF1
UNC2400 (µM)
UNC2400 (µM)
UNC2400 (µM)
RMS-01
CT10
UNC2400 (µM)
UNC2400 (µM)
Figure S2. Dose Response for the EZH2 inhibitor UNC1999 (A) and the inactive analogue UNC2400 (B) in FP-RMS and FN-RMS cell lines combined with DMSO control or 2.5uM ATRA for 6 days. HSSYII and HFF1 used as a positive and negative controls respectively.

## Slide 3
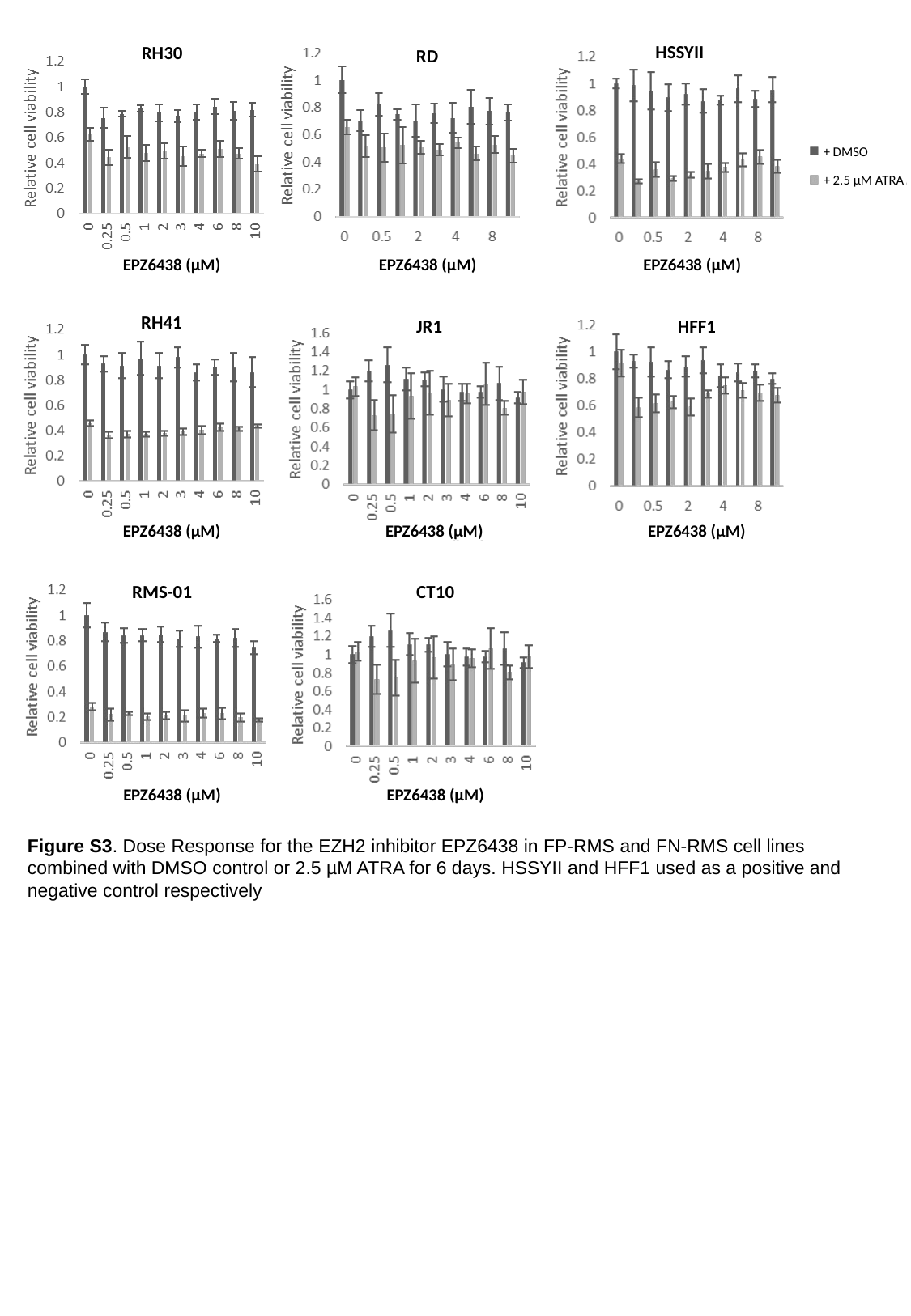

HSSYII
RH30
RD
+ DMSO
+ 2.5 µM ATRA
EPZ6438 (µM)
EPZ6438 (µM)
EPZ6438 (µM)
RH41
JR1
HFF1
EPZ6438 (µM)
EPZ6438 (µM)
EPZ6438 (µM)
RMS-01
CT10
EPZ6438 (µM)
EPZ6438 (µM)
Figure S3. Dose Response for the EZH2 inhibitor EPZ6438 in FP-RMS and FN-RMS cell lines combined with DMSO control or 2.5 µM ATRA for 6 days. HSSYII and HFF1 used as a positive and negative control respectively

## Slide 4
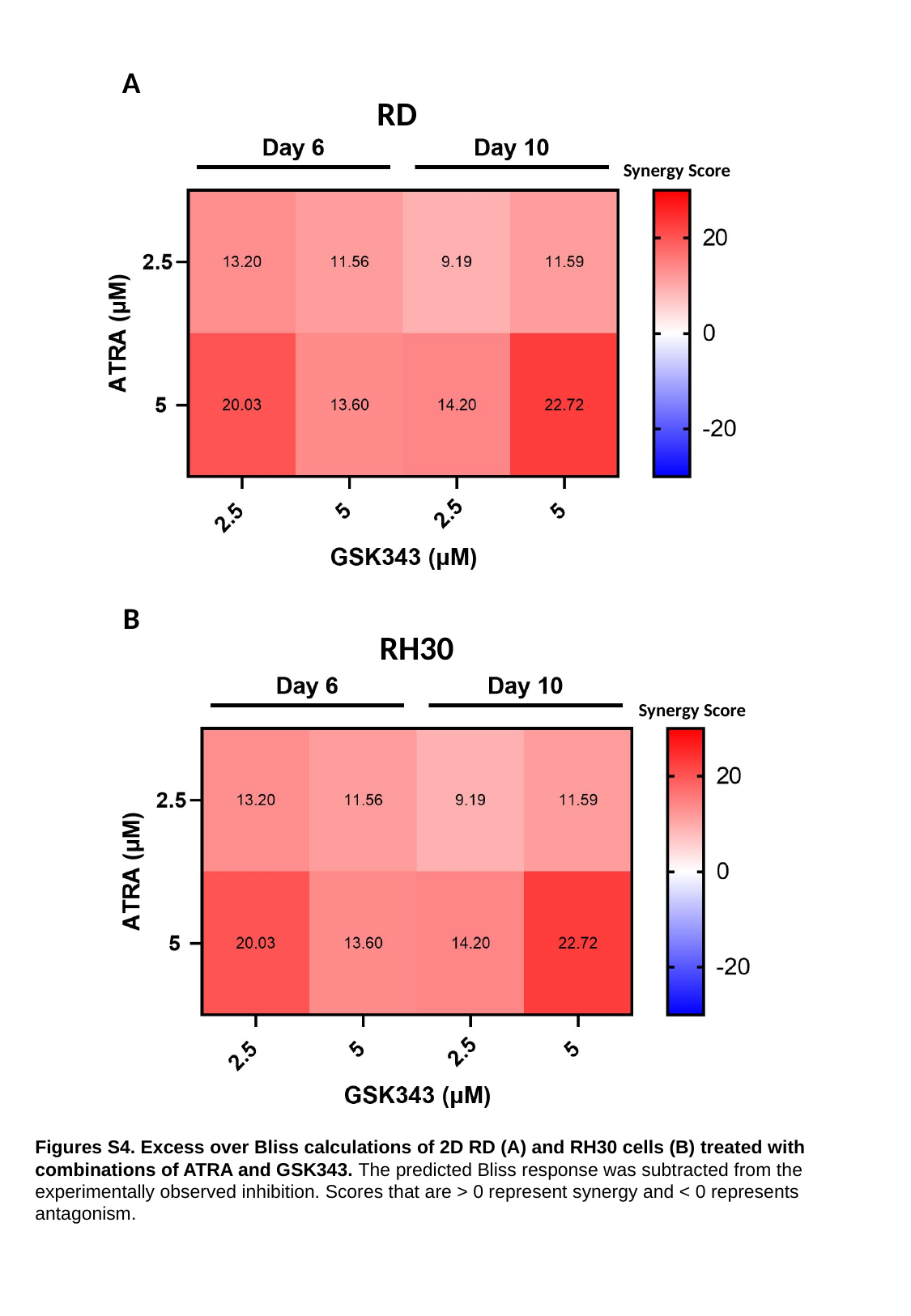

A
RD
Synergy Score
B
RH30
Synergy Score
Figures S4. Excess over Bliss calculations of 2D RD (A) and RH30 cells (B) treated with combinations of ATRA and GSK343. The predicted Bliss response was subtracted from the experimentally observed inhibition. Scores that are > 0 represent synergy and < 0 represents antagonism.

## Slide 5
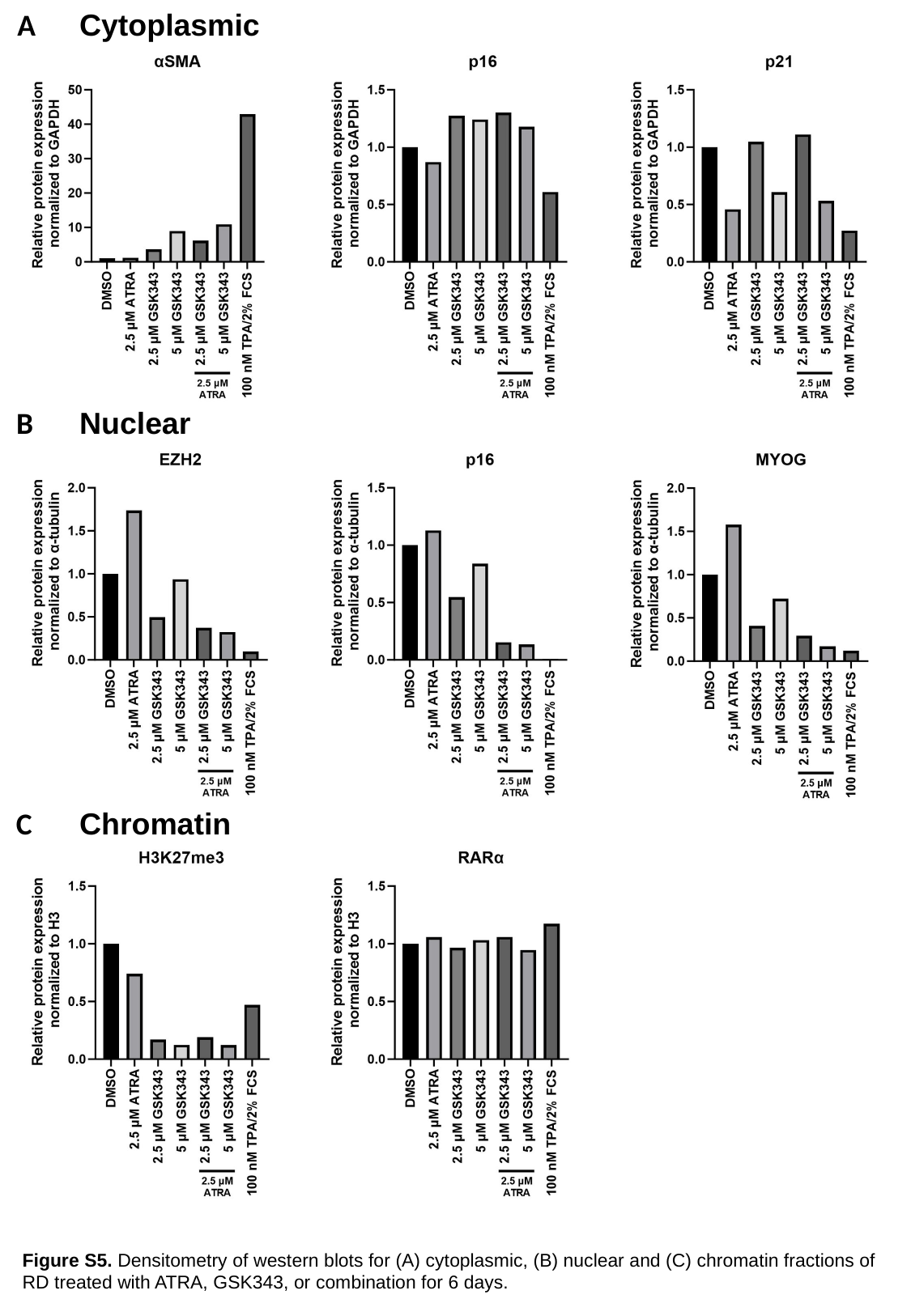

A
Cytoplasmic
B
Nuclear
C
Chromatin
Figure S5. Densitometry of western blots for (A) cytoplasmic, (B) nuclear and (C) chromatin fractions of RD treated with ATRA, GSK343, or combination for 6 days.

## Slide 6
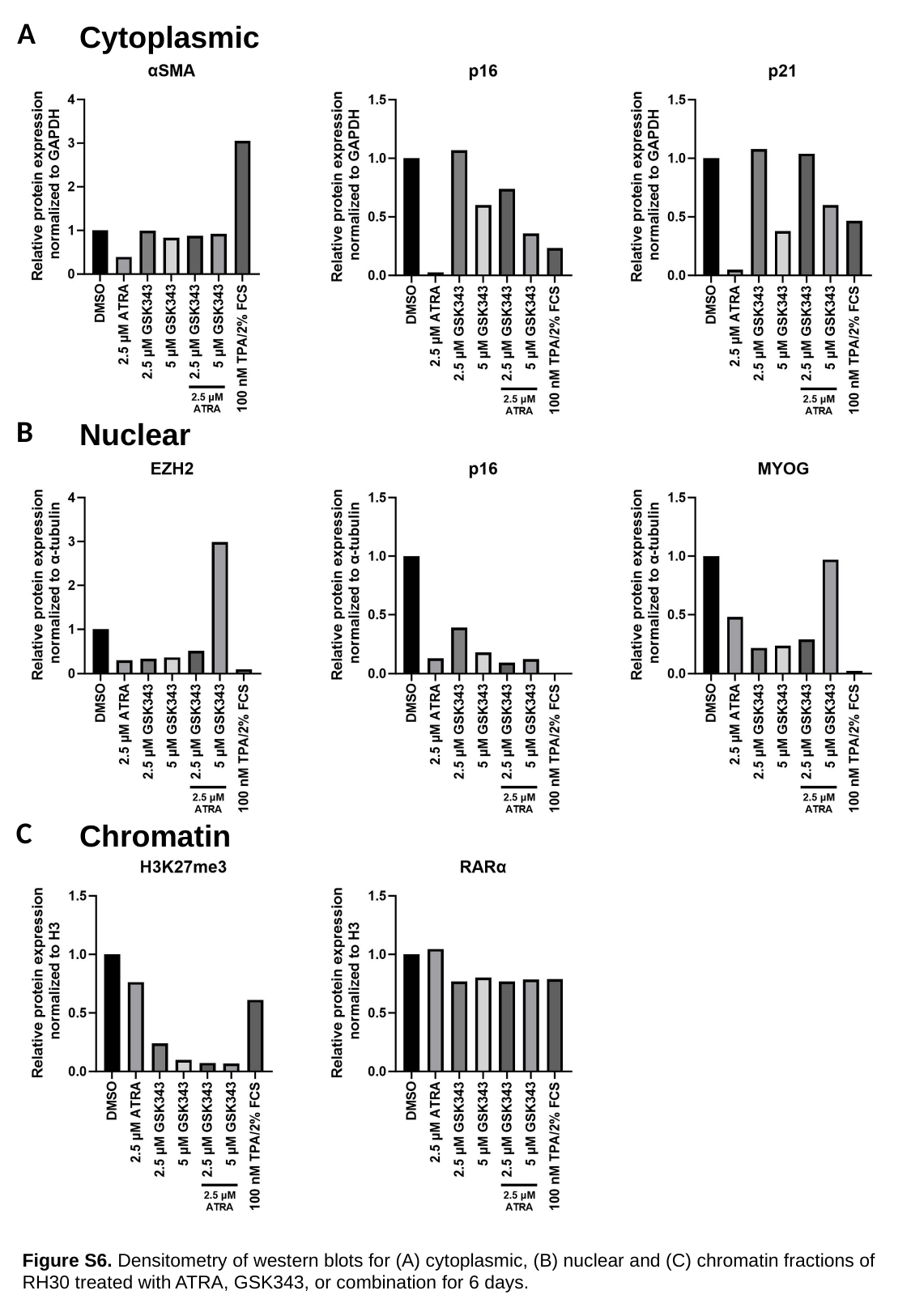

A
Cytoplasmic
B
Nuclear
C
Chromatin
Figure S6. Densitometry of western blots for (A) cytoplasmic, (B) nuclear and (C) chromatin fractions of RH30 treated with ATRA, GSK343, or combination for 6 days.

## Slide 7
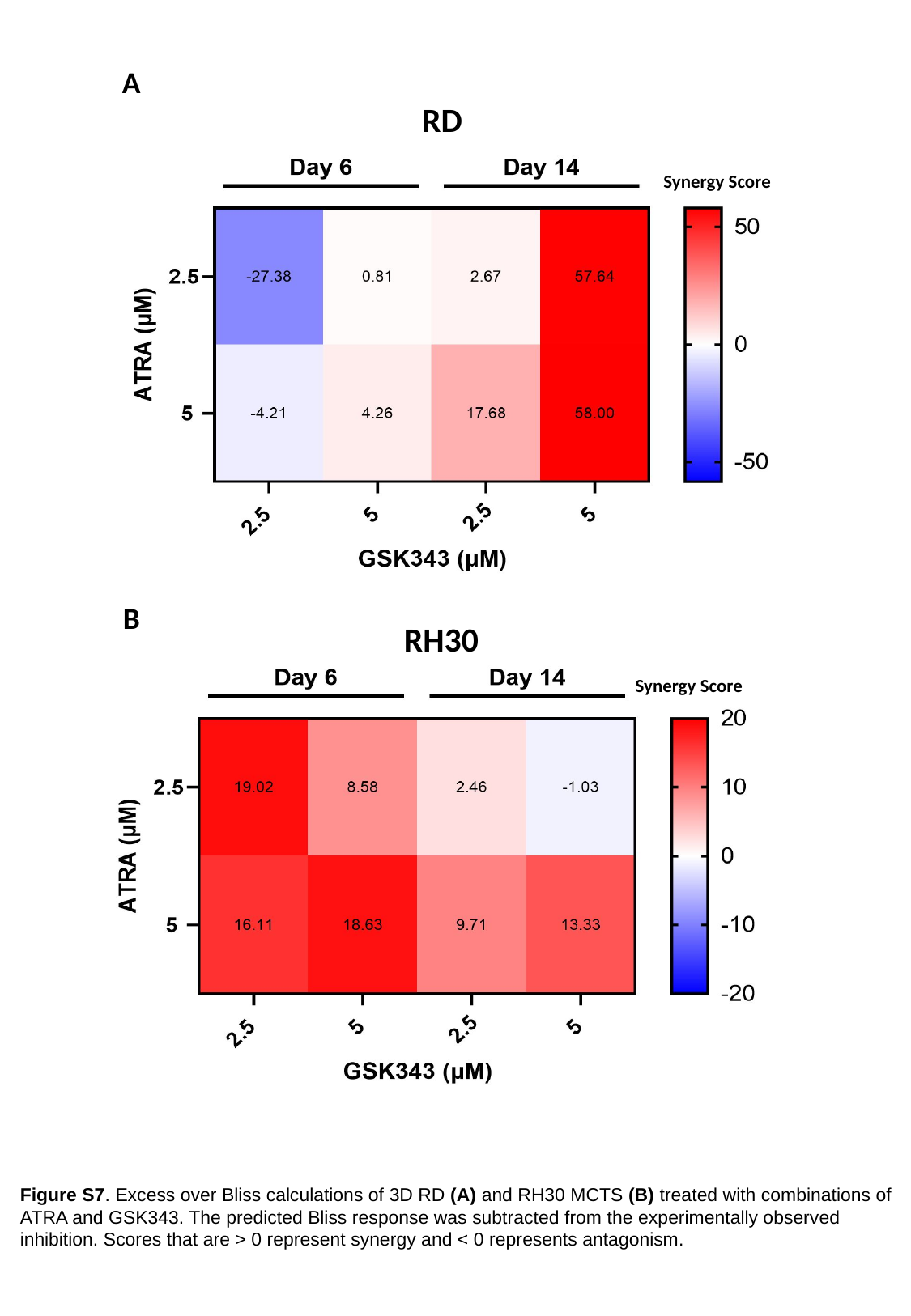

A
RD
Synergy Score
B
RH30
Synergy Score
Figure S7. Excess over Bliss calculations of 3D RD (A) and RH30 MCTS (B) treated with combinations of ATRA and GSK343. The predicted Bliss response was subtracted from the experimentally observed inhibition. Scores that are > 0 represent synergy and < 0 represents antagonism.

## Slide 8
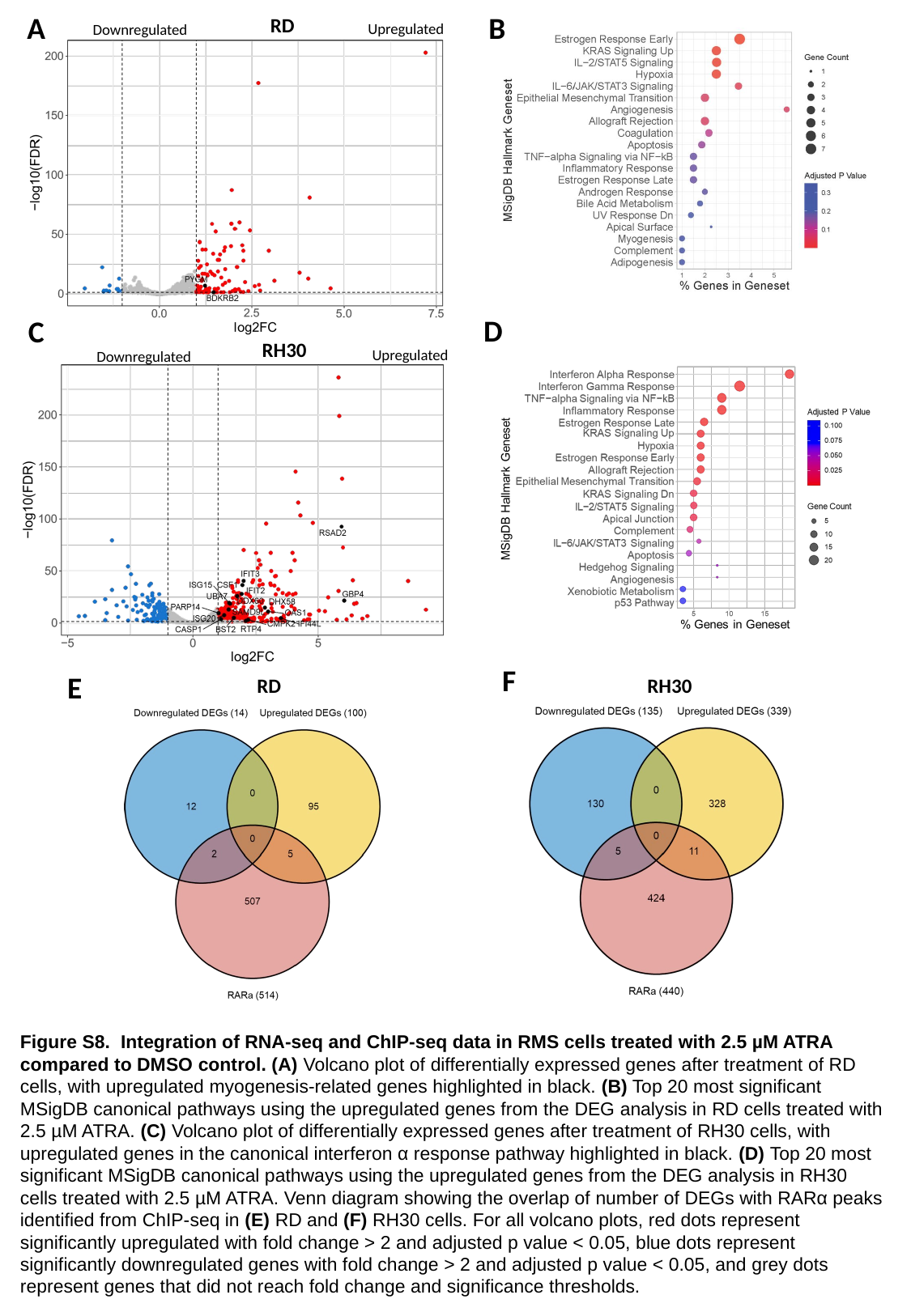

A
B
RD
Upregulated
Downregulated
D
C
RH30
Upregulated
Downregulated
F
E
RD
RH30
Figure S8. Integration of RNA-seq and ChIP-seq data in RMS cells treated with 2.5 µM ATRA compared to DMSO control. (A) Volcano plot of differentially expressed genes after treatment of RD cells, with upregulated myogenesis-related genes highlighted in black. (B) Top 20 most significant MSigDB canonical pathways using the upregulated genes from the DEG analysis in RD cells treated with 2.5 µM ATRA. (C) Volcano plot of differentially expressed genes after treatment of RH30 cells, with upregulated genes in the canonical interferon α response pathway highlighted in black. (D) Top 20 most significant MSigDB canonical pathways using the upregulated genes from the DEG analysis in RH30 cells treated with 2.5 µM ATRA. Venn diagram showing the overlap of number of DEGs with RARα peaks identified from ChIP-seq in (E) RD and (F) RH30 cells. For all volcano plots, red dots represent significantly upregulated with fold change > 2 and adjusted p value < 0.05, blue dots represent significantly downregulated genes with fold change > 2 and adjusted p value < 0.05, and grey dots represent genes that did not reach fold change and significance thresholds.

## Slide 9
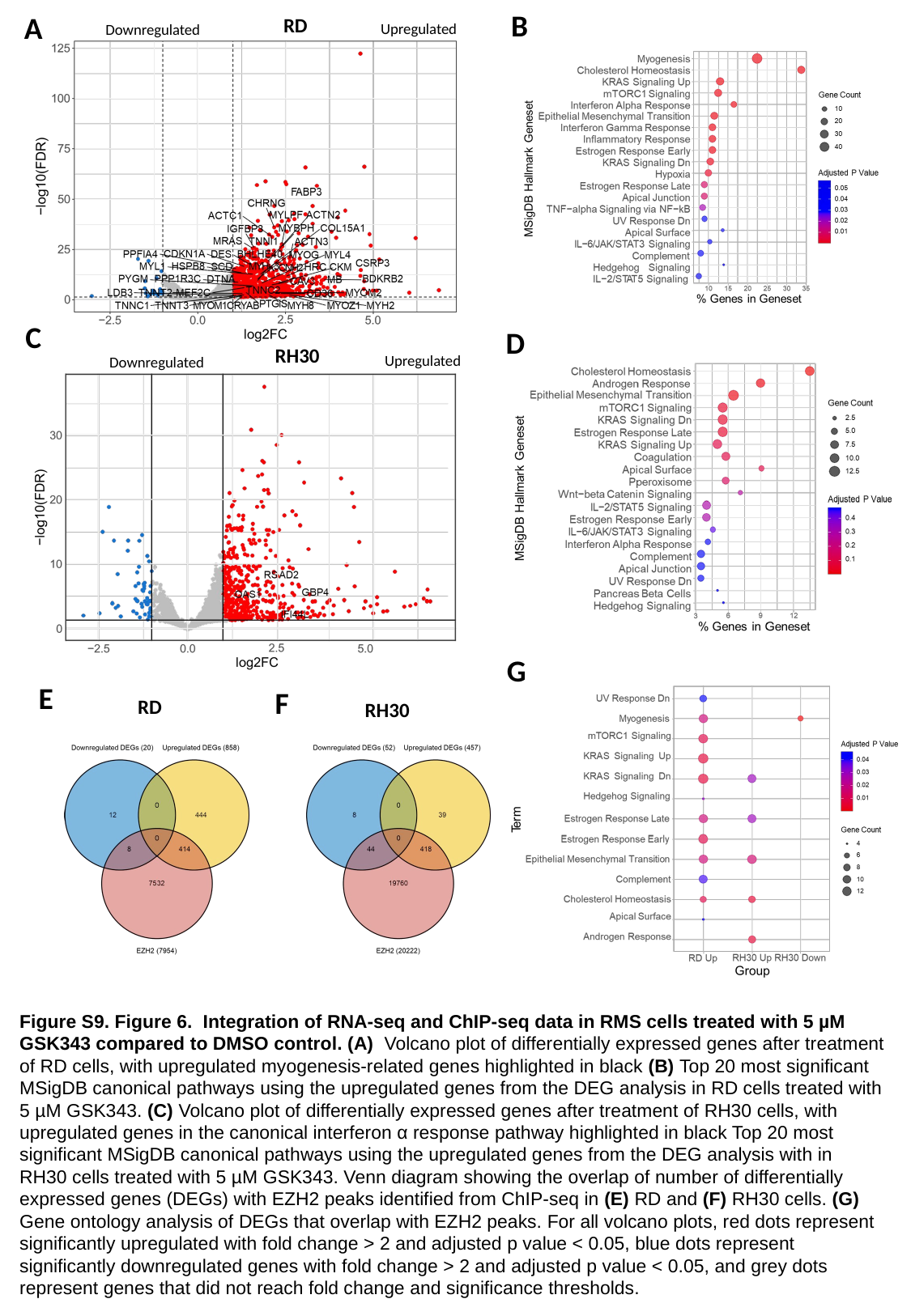

B
A
RD
Upregulated
Downregulated
C
D
RH30
Upregulated
Downregulated
G
E
F
RD
RH30
Figure S9. Figure 6. Integration of RNA-seq and ChIP-seq data in RMS cells treated with 5 µM GSK343 compared to DMSO control. (A) Volcano plot of differentially expressed genes after treatment of RD cells, with upregulated myogenesis-related genes highlighted in black (B) Top 20 most significant MSigDB canonical pathways using the upregulated genes from the DEG analysis in RD cells treated with 5 µM GSK343. (C) Volcano plot of differentially expressed genes after treatment of RH30 cells, with upregulated genes in the canonical interferon α response pathway highlighted in black Top 20 most significant MSigDB canonical pathways using the upregulated genes from the DEG analysis with in RH30 cells treated with 5 µM GSK343. Venn diagram showing the overlap of number of differentially expressed genes (DEGs) with EZH2 peaks identified from ChIP-seq in (E) RD and (F) RH30 cells. (G) Gene ontology analysis of DEGs that overlap with EZH2 peaks. For all volcano plots, red dots represent significantly upregulated with fold change > 2 and adjusted p value < 0.05, blue dots represent significantly downregulated genes with fold change > 2 and adjusted p value < 0.05, and grey dots represent genes that did not reach fold change and significance thresholds.

## Slide 10
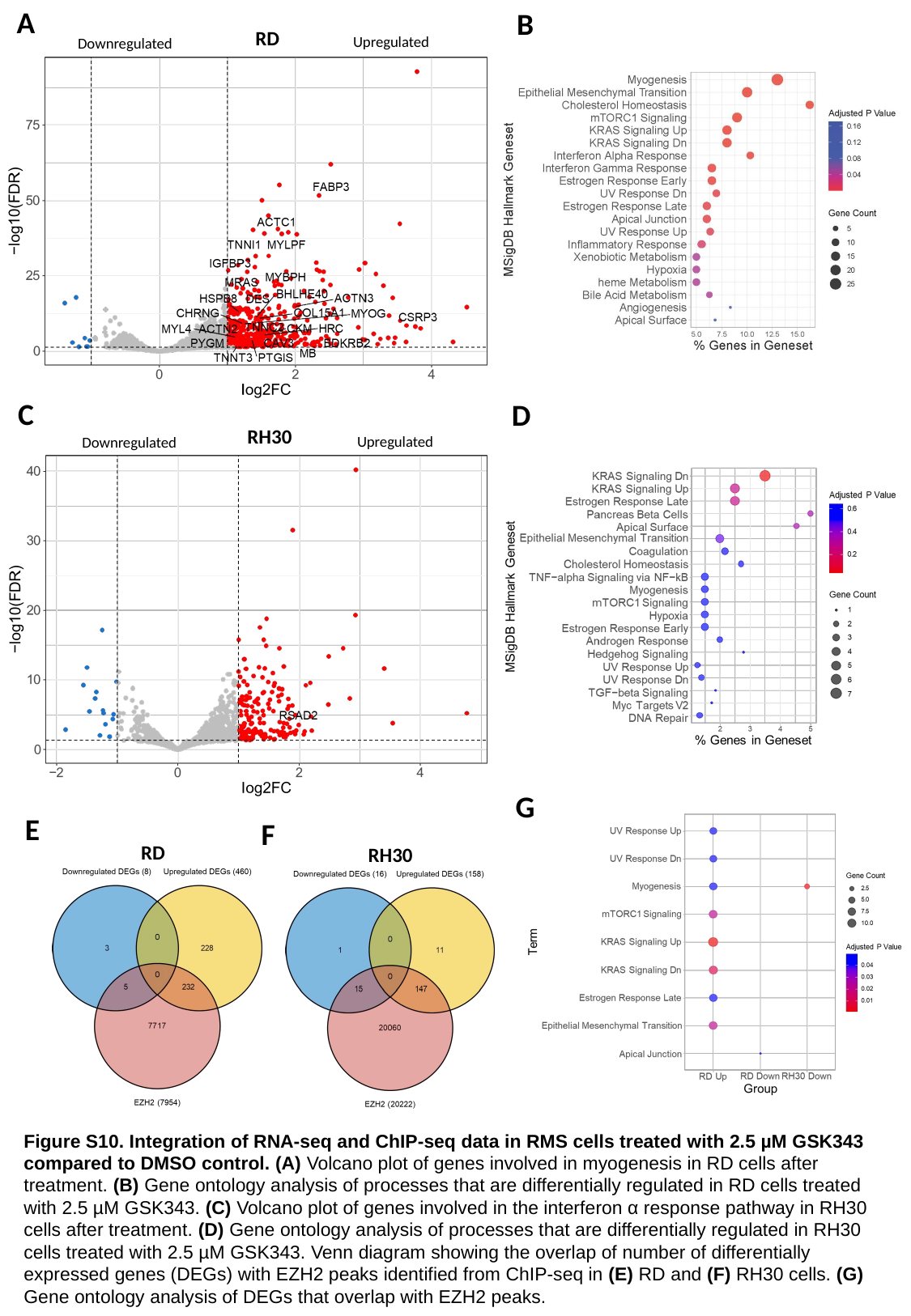

A
B
RD
Upregulated
Downregulated
C
D
RH30
Upregulated
Downregulated
G
E
F
RD
RH30
Figure S10. Integration of RNA-seq and ChIP-seq data in RMS cells treated with 2.5 µM GSK343 compared to DMSO control. (A) Volcano plot of genes involved in myogenesis in RD cells after treatment. (B) Gene ontology analysis of processes that are differentially regulated in RD cells treated with 2.5 µM GSK343. (C) Volcano plot of genes involved in the interferon α response pathway in RH30 cells after treatment. (D) Gene ontology analysis of processes that are differentially regulated in RH30 cells treated with 2.5 µM GSK343. Venn diagram showing the overlap of number of differentially expressed genes (DEGs) with EZH2 peaks identified from ChIP-seq in (E) RD and (F) RH30 cells. (G) Gene ontology analysis of DEGs that overlap with EZH2 peaks.

## Slide 11
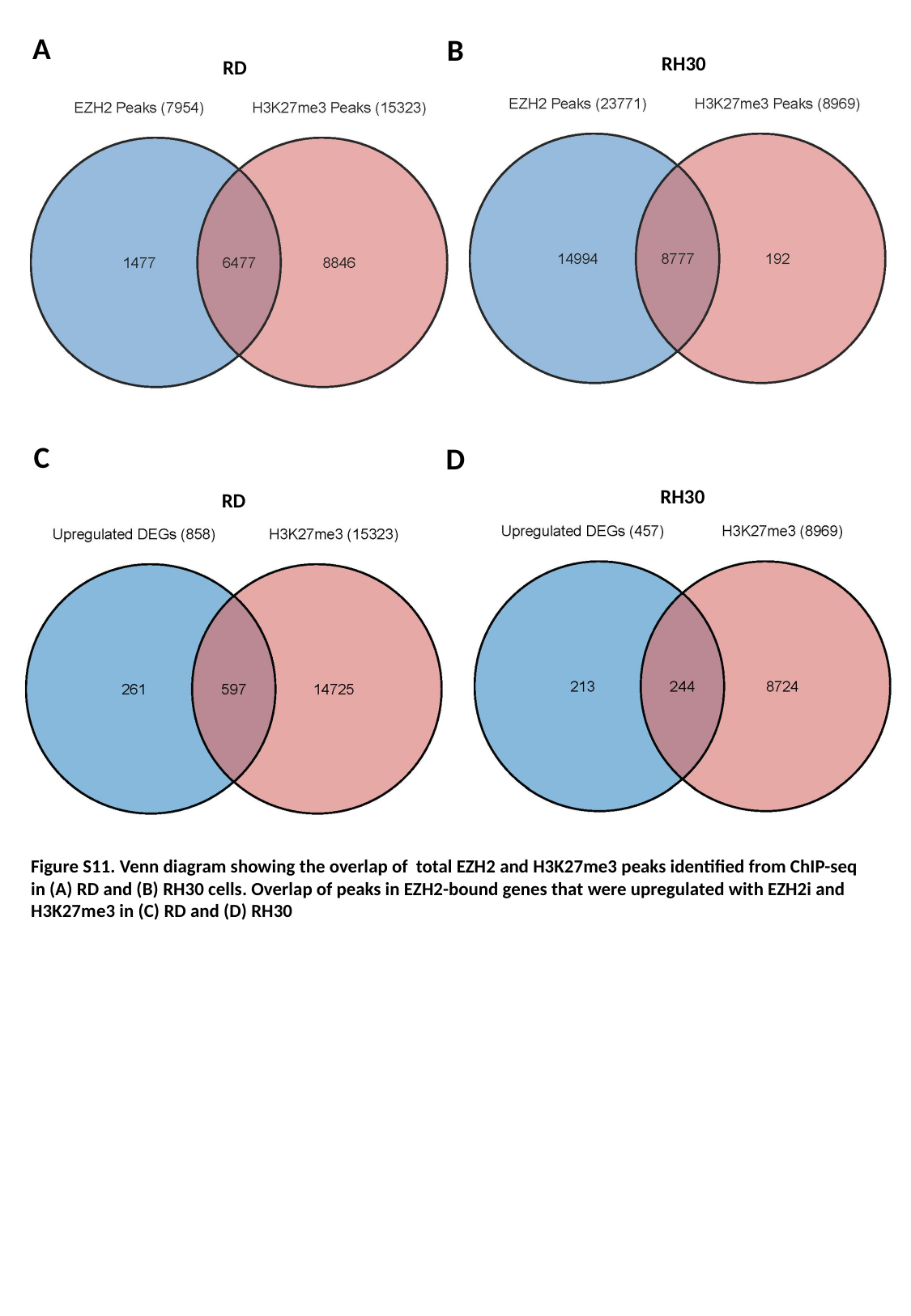

A
B
RH30
RD
C
D
RH30
RD
Figure S11. Venn diagram showing the overlap of total EZH2 and H3K27me3 peaks identified from ChIP-seq in (A) RD and (B) RH30 cells. Overlap of peaks in EZH2-bound genes that were upregulated with EZH2i and H3K27me3 in (C) RD and (D) RH30

## Slide 12
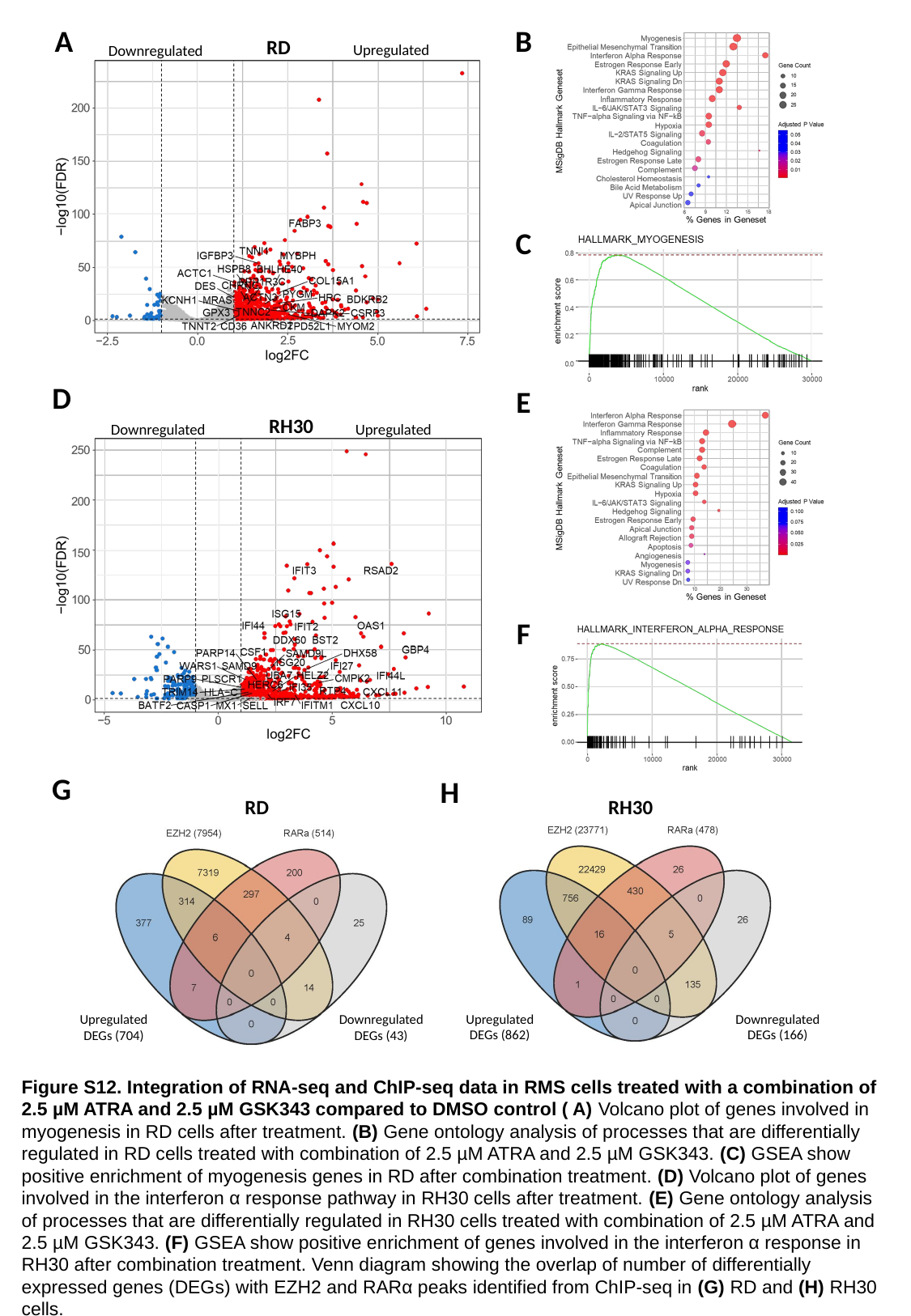

A
B
RD
Upregulated
Downregulated
C
D
E
RH30
Upregulated
Downregulated
F
G
H
RD
RH30
Upregulated DEGs (862)
Downregulated DEGs (166)
Upregulated DEGs (704)
Downregulated DEGs (43)
Figure S12. Integration of RNA-seq and ChIP-seq data in RMS cells treated with a combination of 2.5 µM ATRA and 2.5 µM GSK343 compared to DMSO control ( A) Volcano plot of genes involved in myogenesis in RD cells after treatment. (B) Gene ontology analysis of processes that are differentially regulated in RD cells treated with combination of 2.5 µM ATRA and 2.5 µM GSK343. (C) GSEA show positive enrichment of myogenesis genes in RD after combination treatment. (D) Volcano plot of genes involved in the interferon α response pathway in RH30 cells after treatment. (E) Gene ontology analysis of processes that are differentially regulated in RH30 cells treated with combination of 2.5 µM ATRA and 2.5 µM GSK343. (F) GSEA show positive enrichment of genes involved in the interferon α response in RH30 after combination treatment. Venn diagram showing the overlap of number of differentially expressed genes (DEGs) with EZH2 and RARα peaks identified from ChIP-seq in (G) RD and (H) RH30 cells.

## Slide 13
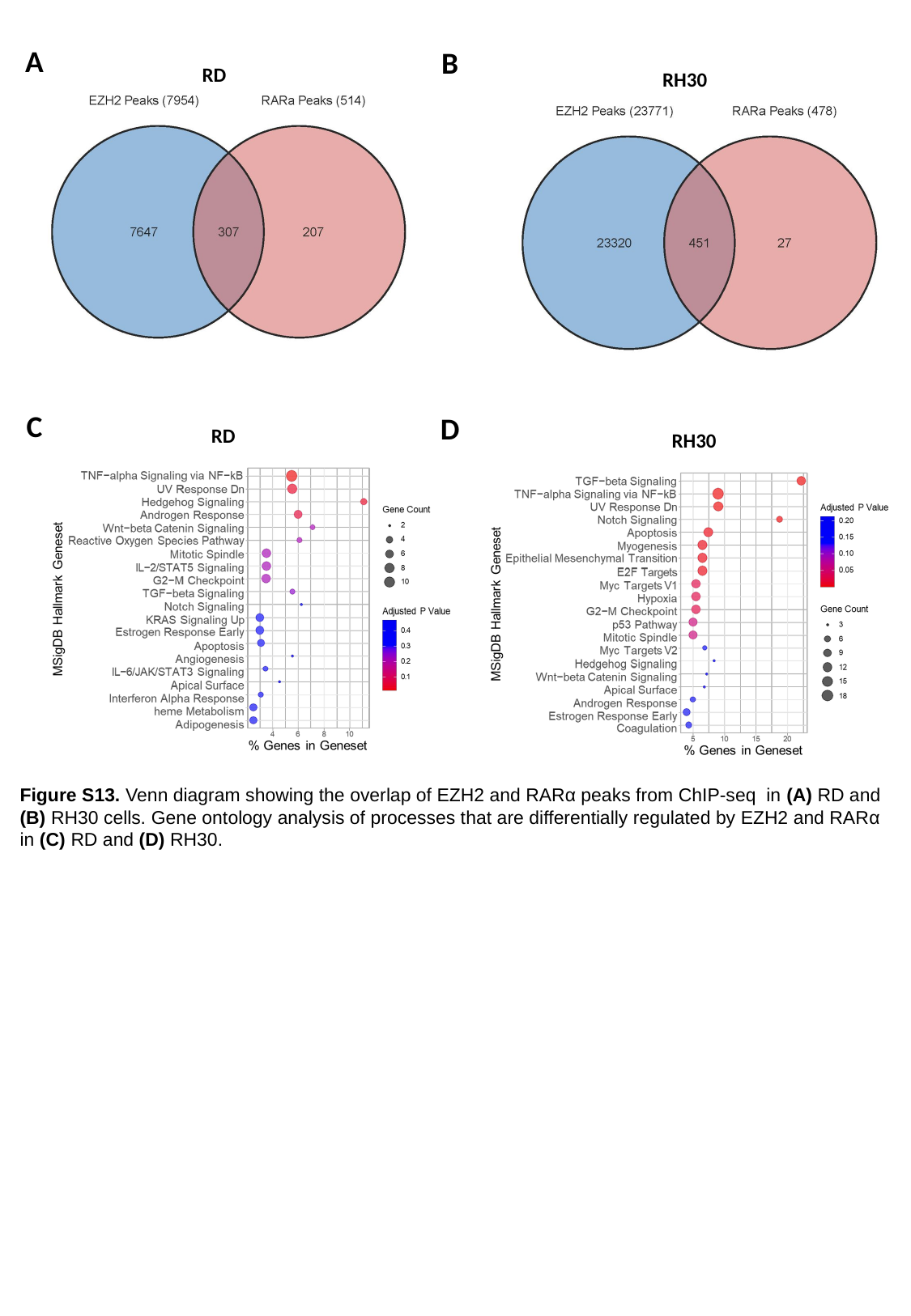

A
B
RD
RH30
C
D
RD
RH30
Figure S13. Venn diagram showing the overlap of EZH2 and RARα peaks from ChIP-seq in (A) RD and (B) RH30 cells. Gene ontology analysis of processes that are differentially regulated by EZH2 and RARα in (C) RD and (D) RH30.

## Slide 14
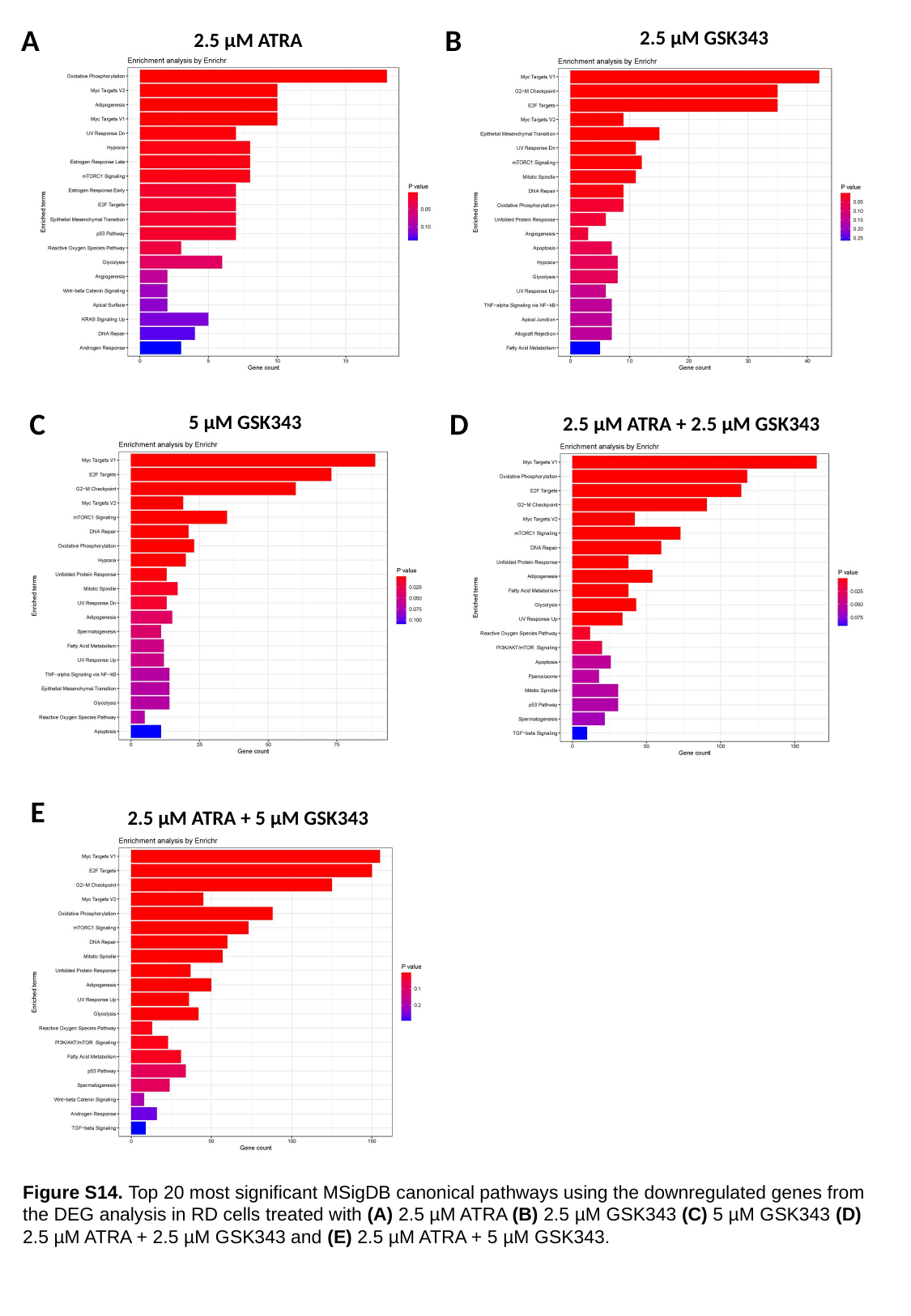

A
B
2.5 µM GSK343
2.5 µM ATRA
C
D
5 µM GSK343
2.5 µM ATRA + 2.5 µM GSK343
E
2.5 µM ATRA + 5 µM GSK343
Figure S14. Top 20 most significant MSigDB canonical pathways using the downregulated genes from the DEG analysis in RD cells treated with (A) 2.5 µM ATRA (B) 2.5 µM GSK343 (C) 5 µM GSK343 (D) 2.5 µM ATRA + 2.5 µM GSK343 and (E) 2.5 µM ATRA + 5 µM GSK343.

## Slide 15
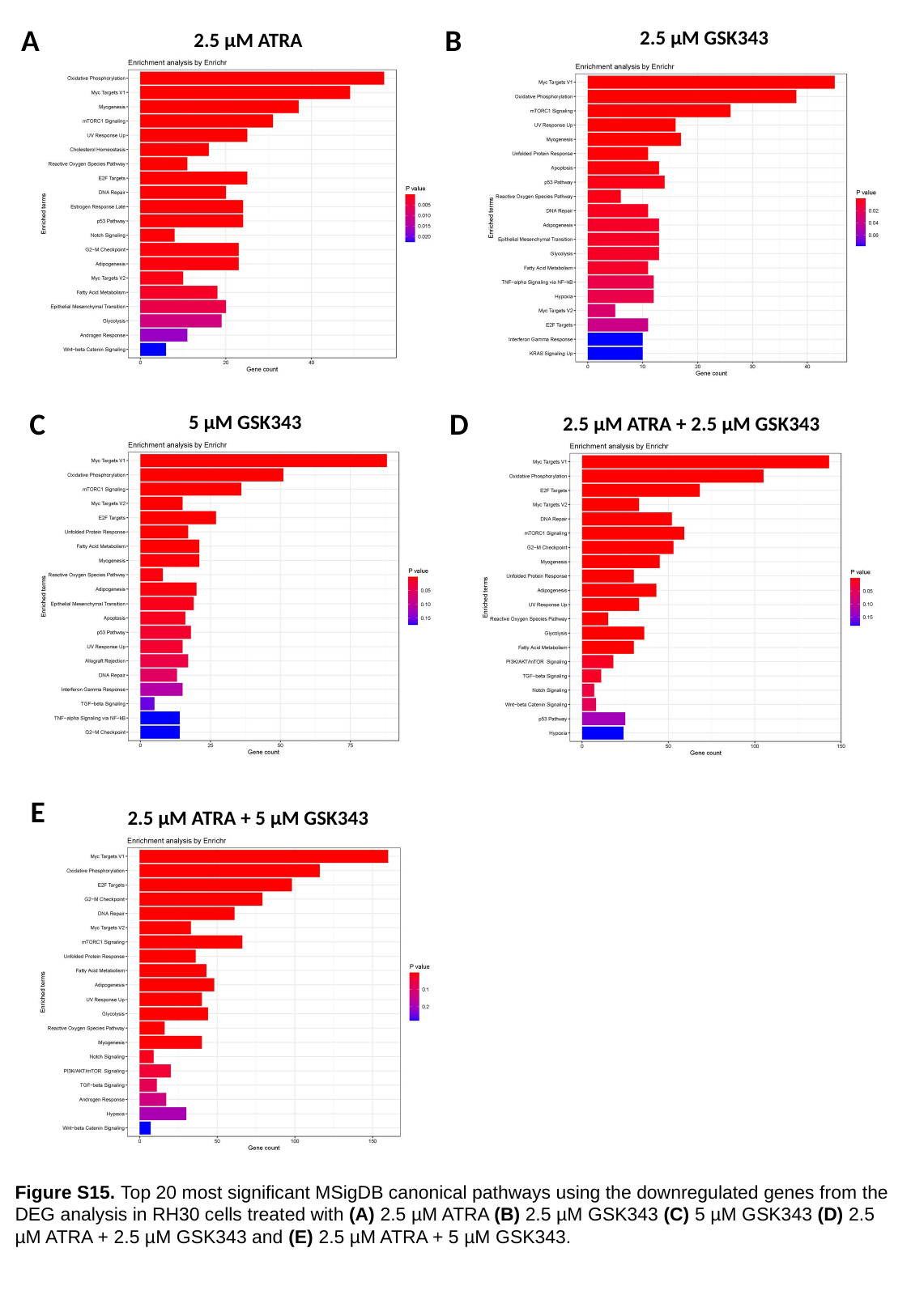

A
B
2.5 µM GSK343
2.5 µM ATRA
C
D
5 µM GSK343
2.5 µM ATRA + 2.5 µM GSK343
E
2.5 µM ATRA + 5 µM GSK343
Figure S15. Top 20 most significant MSigDB canonical pathways using the downregulated genes from the DEG analysis in RH30 cells treated with (A) 2.5 µM ATRA (B) 2.5 µM GSK343 (C) 5 µM GSK343 (D) 2.5 µM ATRA + 2.5 µM GSK343 and (E) 2.5 µM ATRA + 5 µM GSK343.

## Slide 16
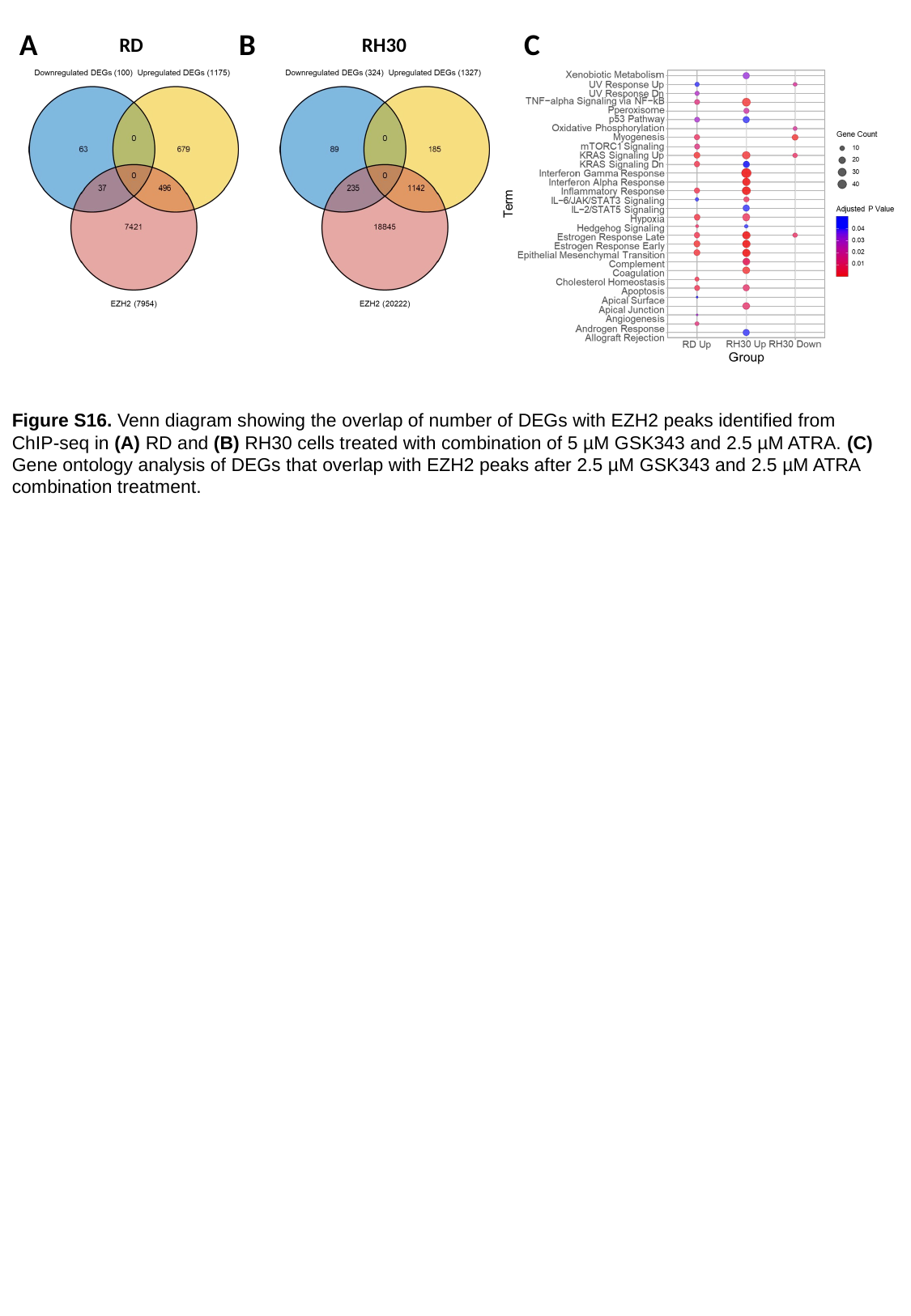

A
B
C
RD
RH30
Figure S16. Venn diagram showing the overlap of number of DEGs with EZH2 peaks identified from ChIP-seq in (A) RD and (B) RH30 cells treated with combination of 5 µM GSK343 and 2.5 µM ATRA. (C) Gene ontology analysis of DEGs that overlap with EZH2 peaks after 2.5 µM GSK343 and 2.5 µM ATRA combination treatment.
